# Supplementary material for: Integrative multi-omics analysis of muscle-invasive bladder cancer identifies prognostic biomarkers for frontline chemotherapy and immunotherapy
Source: Commun Biol. 2020 Dec 17;3:784. doi: 10.1038/s42003-020-01491-2 (PMC7746703; doi:10.1038/s42003-020-01491-2)
Supplement: Supplementary file 2 — Supplementary Information [file 42003_2020_1491_MOESM2_ESM.pdf]

Integrative multi-omics analysis of muscle-invasive bladder cancer identifies prognostic biomarkers for frontline chemotherapy and immunotherapy

Qianxing Mo[1*], Roger Li[2], Dennis O. Adeegbe[3], Guang Peng[4], Keith Syson Chan[5]

1. Department of Biostatistics & Bioinformatics, H. Lee Moffitt Cancer Center & Research Institute, Tampa, FL 33612
2. Department of Genitourinary Oncology, H. Lee Moffitt Cancer Center & Research Institute, Tampa, FL, 33612, USA.
3. Department of Immunology, H. Lee Moffitt Cancer Center & Research Institute, Tampa, FL, 33612, USA.
4. Department of Clinical Cancer Prevention, The University of Texas MD Anderson Cancer Center, Houston, Texas, 77030, USA
5. Department of Pathology and Samuel Oschin Comprehensive Cancer Institute, Cedars-Sinai Medical Center, Los Angeles, CA 90048, USA

* Corresponding author

Qianxing Mo, Ph.D.

Department of Biostatistics & Bioinformatics

H. Lee Moffitt Cancer Center & Research Institute

12902 Magnolia Drive, Tampa, FL 33612

Phone: 813-745-8835

Email: qianxing.mo@moffitt.org

Supplementary Figures 1-10 (pages 1-8)

Supplementary Tables 1-2 (pages 9-10)

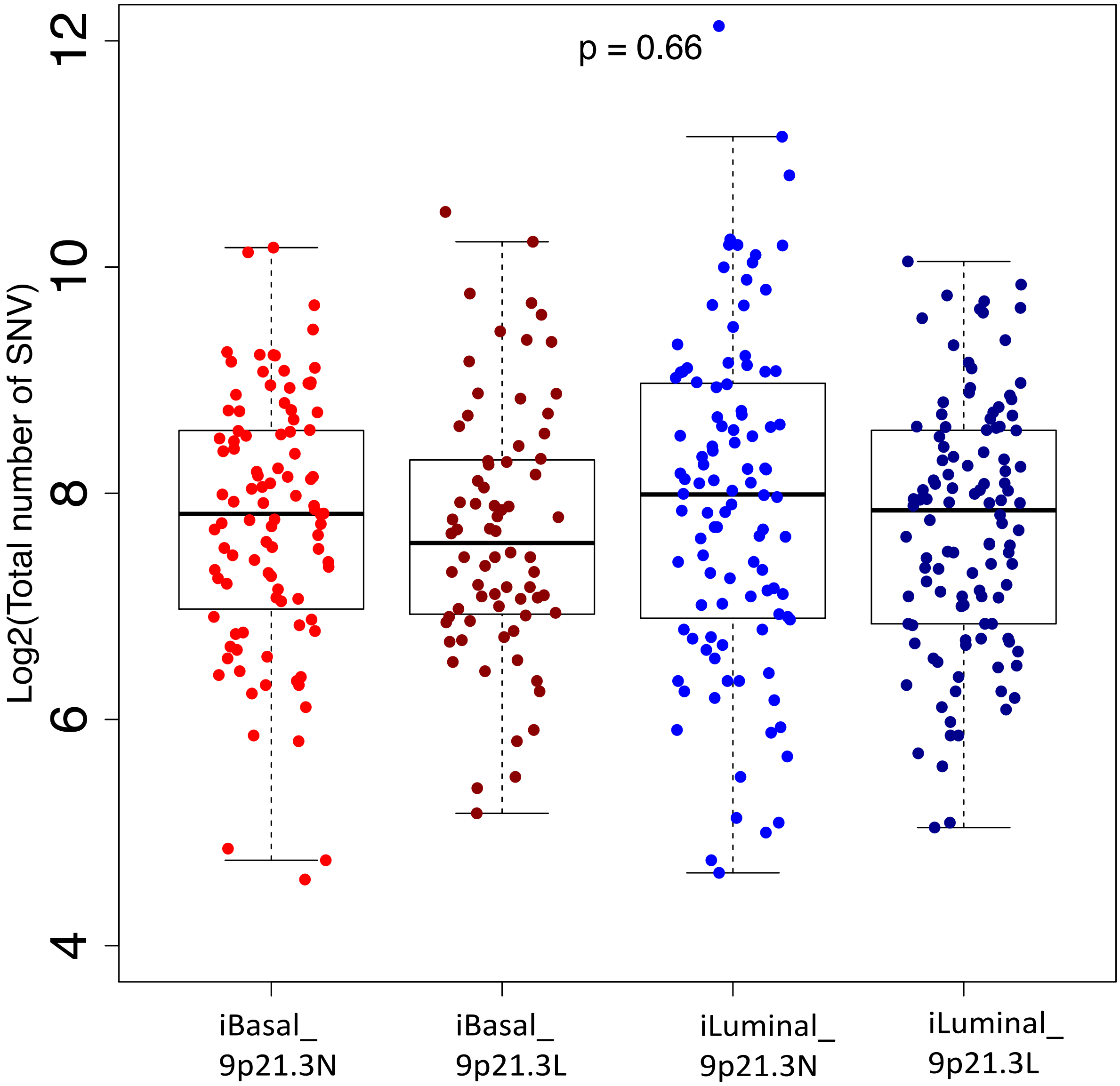

Supplementary Figure 1. Total number of single nucleotide variants (SNV) distributed in the iSubtypes of the TCGA MIBC samples. ANOVA was used for comparison among the subtypes.

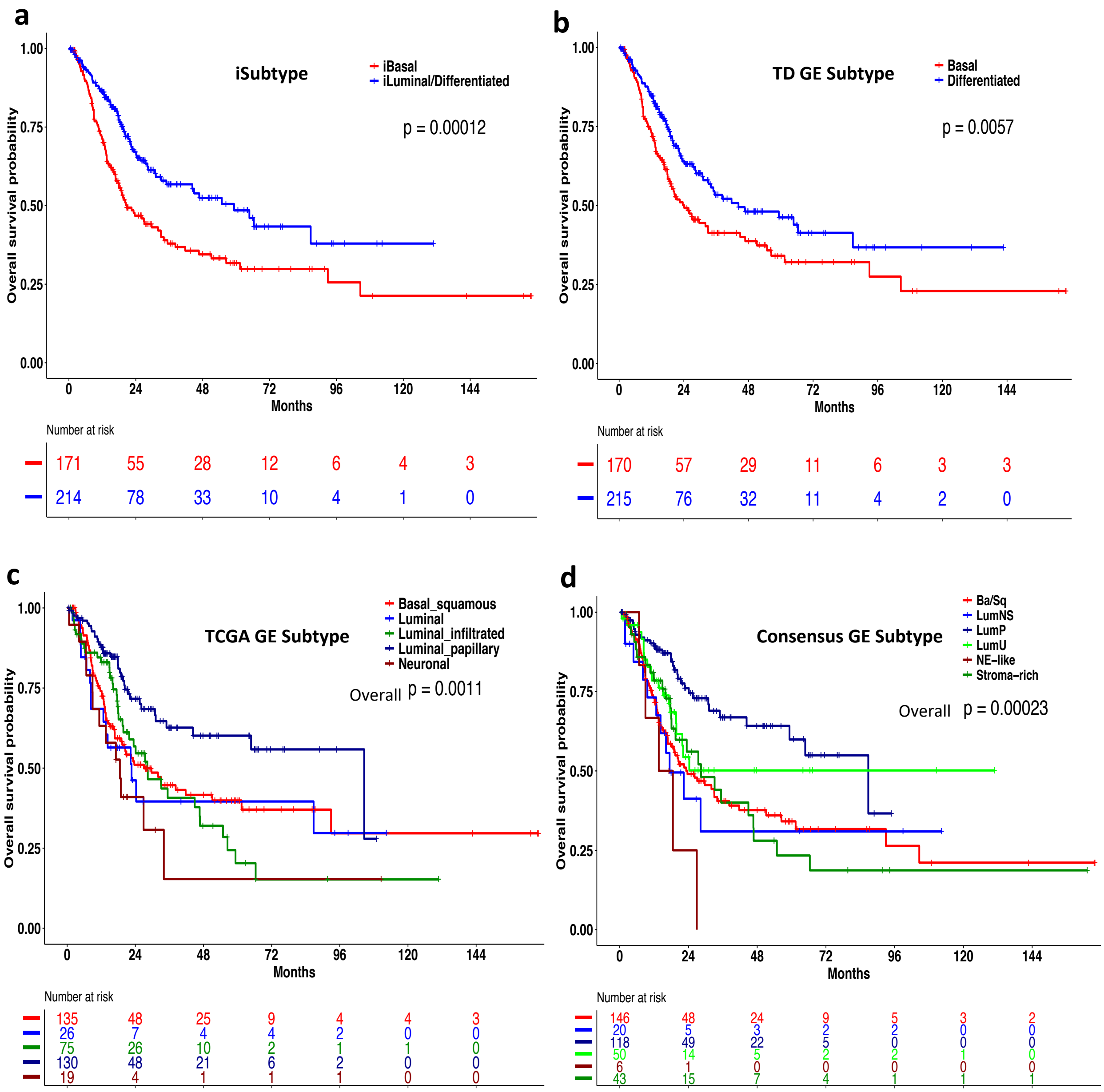

Supplementary Figure 2. Comparison of iSubtypes with other gene expression (GE) based subtypes in the TCGA MIBC cohort (n=385) including 10 patients with history of chemotherapy. **(a)** Patient overall survival stratified by iBasal and iLuminal/Differentiated sub types. **(b)** Patient overall survival stratified by the basal and differentiated subtypes defined by the TD GE signature. **(c)** Patient overall survival stratified by the TCGA GE subtypes. **(d)** Patient overall survival stratified by the consensus GE subtypes. Ba/Sq: basal/squamous; LumNS= luminal nonspecified; LumP= luminal papillary; LumU= luminal unstable; NE= neuroendocrine. Log-rank test was used to compare subtype-specific survival curves.

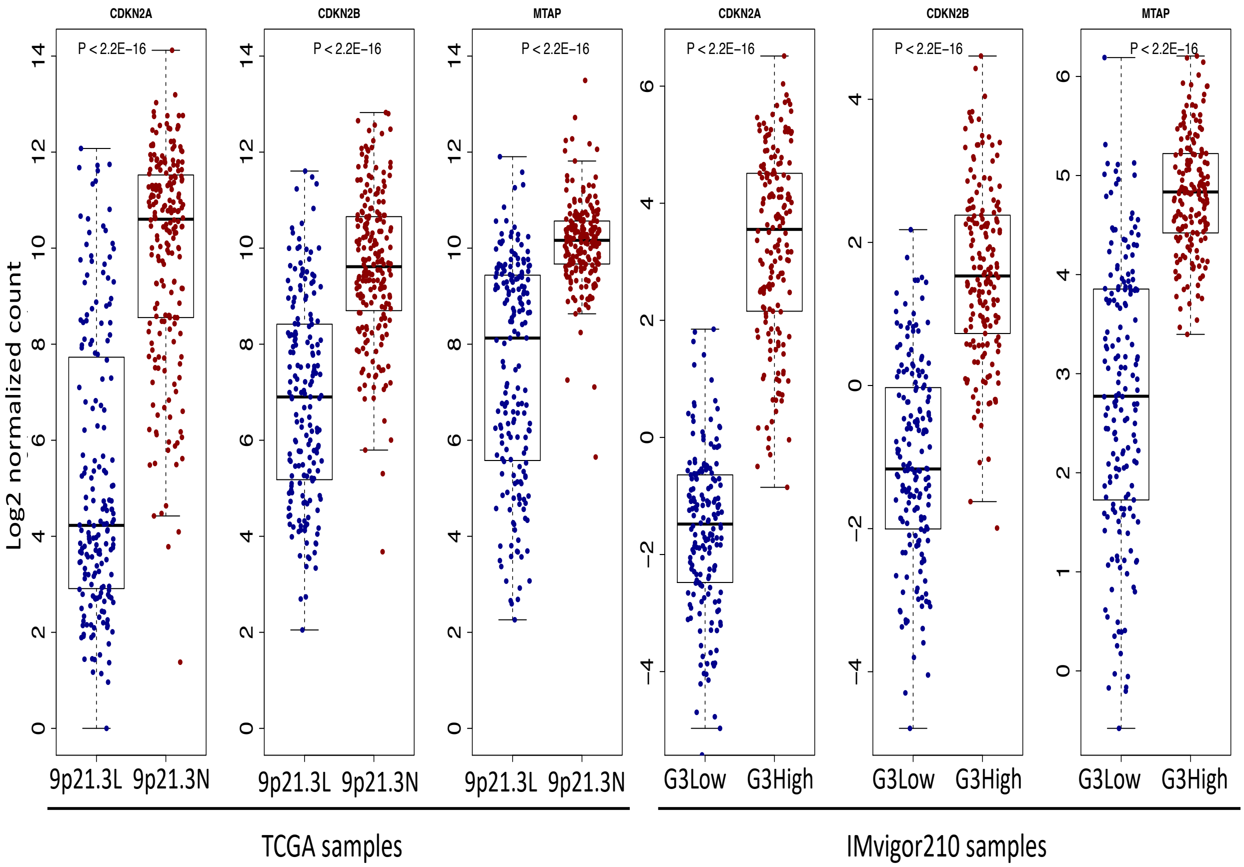

Supplementary Figure 3. CDKN2A/2B and MTAP expression in the TCGA and IMvigor210 samples. G3High/Low corresponds to chr9 p21.3N/L, respectively. Two sa mple t-test was used to compare gene expression between the groups.

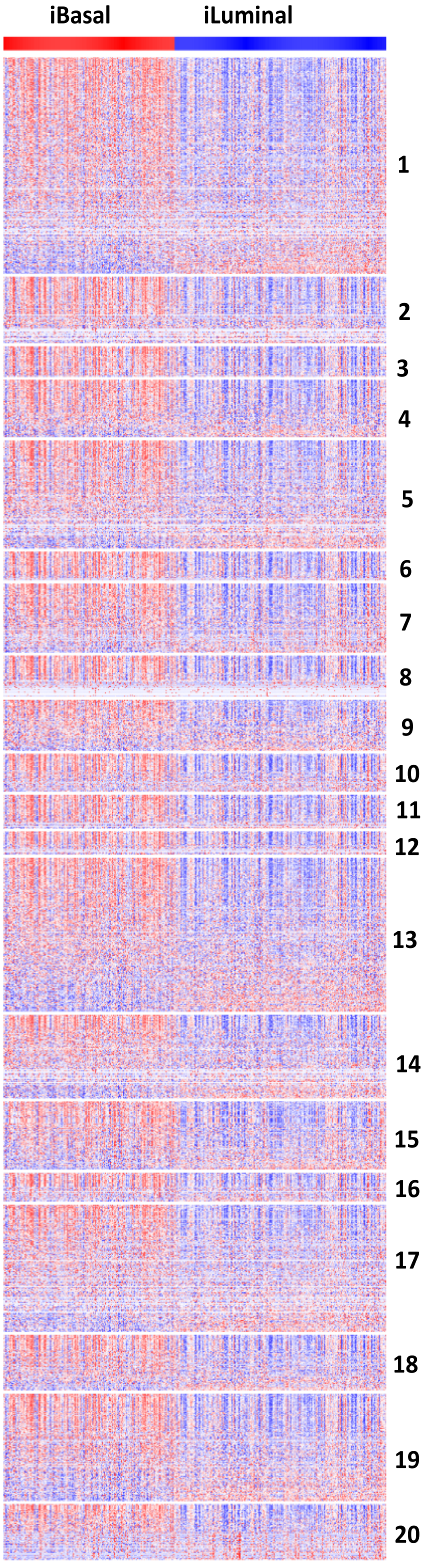

ID	KEGG PATHWAY	SIZE	FDR
1	CYTOKINE_CYTOKINE_RECEPTOR_INTERACTION	264	<0.0001
2	ANTIGEN_PROCESSING_AND_PRESENTATION	81	<0.0001
3	GRAFT_VERSUS_HOST_DISEASE	37	<0.0001
4	LEISHMANIA_INFECTION	70	<0.0001
5	NATURAL_KILLER_CELL_MEDIATED_CYTOTOXICITY	132	<0.0001
6	ALLOGRAFT_REJECTION	35	<0.0001
7	HEMATOPOIETIC_CELL_LINEAGE	84	<0.0001
8	AUTOIMMUNE_THYROID_DISEASE	50	<0.0001
9	NOD_LIKE_RECEPTOR_SIGNALING_PATHWAY	62	<0.0001
10	INTESTINAL_IMMUNE_NETWORK_FOR_IGA_PRODUCTION	46	<0.0001
11	TYPE_I_DIABETES_MELLITUS	41	<0.0001
12	ASTHMA	28	<0.0001
13	CHEMOKINE_SIGNALING_PATHWAY	188	<0.0001
14	TOLL_LIKE_RECEPTOR_SIGNALING_PATHWAY	102	<0.0001
15	ECM_RECEPTOR_INTERACTION	83	<0.0001
16	PRIMARY_IMMUNODEFICIENCY	35	<0.0001
17	JAK_STAT_SIGNALING_PATHWAY	155	<0.0001
18	VIRAL_MYOCARDITIS	68	1.41E-04
19	CELL_ADHESION_MOLECULES_CAMS	131	1.68E-04
20	COMPLEMENT_AND_COAGULATION_CASCADES	68	2.16E-04

Supplementary Figure 4. Top up-regulated KEGG pathways in the iBasal subtype of the TCGA MIBC samples. Gene-based permutation test was performed to calculate the FDR.

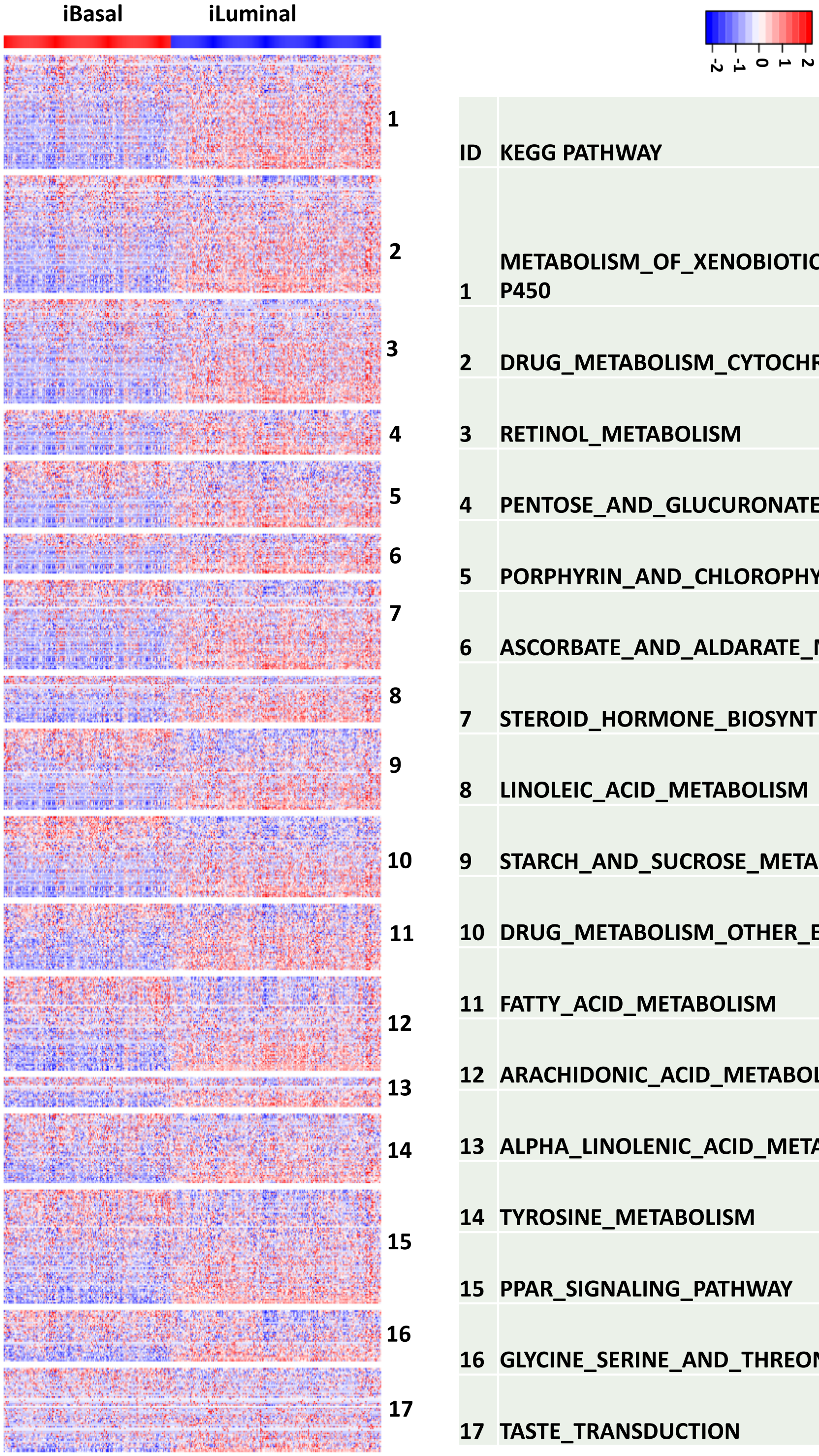

ID	KEGG PATHWAY	SIZE	FDR
1	METABOLISM_OF_XENOBIOTICS_BY_CYTOCHROME_P450	69	<0.0001
2	DRUG_METABOLISM_CYTOCHROME_P450	71	<0.0001
3	RETINOL_METABOLISM	63	<0.0001
4	PENTOSE_AND_GLUCURONATE_INTERCONVERSIONS	27	<0.0001
5	PORPHYRIN_AND_CHLOROPHYLL_METABOLISM	40	<0.0001
6	ASCORBATE_AND_ALDARATE_METABOLISM	24	<0.0001
7	STEROID_HORMONE_BIOSYNTHESIS	54	<0.0001
8	LINOLEIC_ACID_METABOLISM	28	1.44E-04
9	STARCH_AND_SUCROSE_METABOLISM	49	3.35E-04
10	DRUG_METABOLISM_OTHER_ENZYMES	49	6.48E-04
11	FATTY_ACID_METABOLISM	40	0.0028
12	ARACHIDONIC_ACID_METABOLISM	57	0.0054
13	ALPHA_LINOLENIC_ACID_METABOLISM	18	0.031
14	TYROSINE_METABOLISM	42	0.036
15	PPAR_SIGNALING_PATHWAY	69	0.04
16	GLYCINE_SERINE_AND_THREONINE_METABOLISM	31	0.045
17	TASTE_TRANSDUCTION	51	0.05

Supplementary Figure 5. Top up-regulated KEGG pathways in the iLuminal subtype of the TCGA MIBC samples. Gene-based permutation test was performed to calculate the FDR.

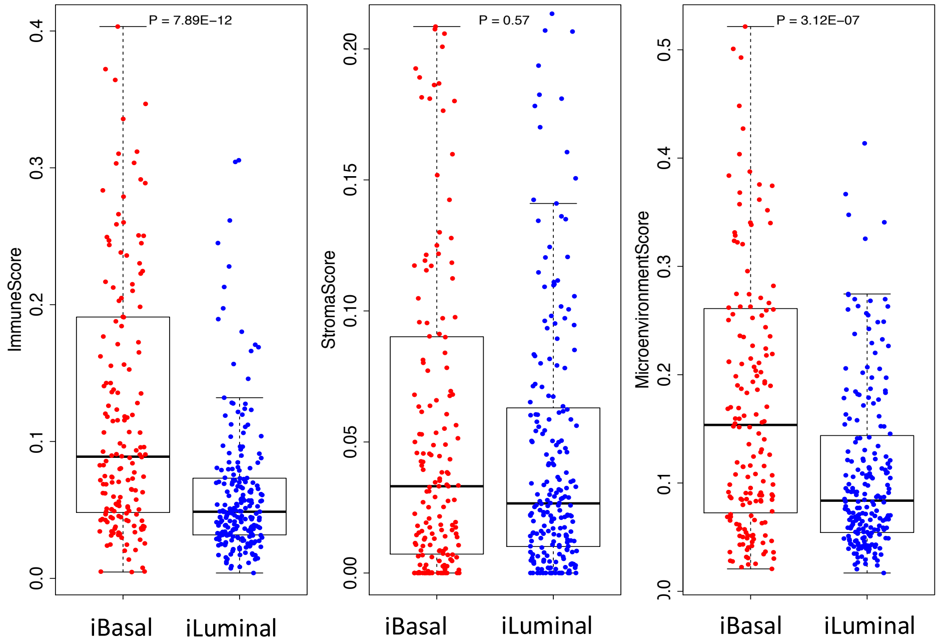

Supplementary Figure 6. Immune, stroma and microenvironment scores in the iBasal and iLuminal/iDifferentiated subtypes of the TCGA MIBC samples. Wilcoxon rank sum test was used for comparison between the subtypes.

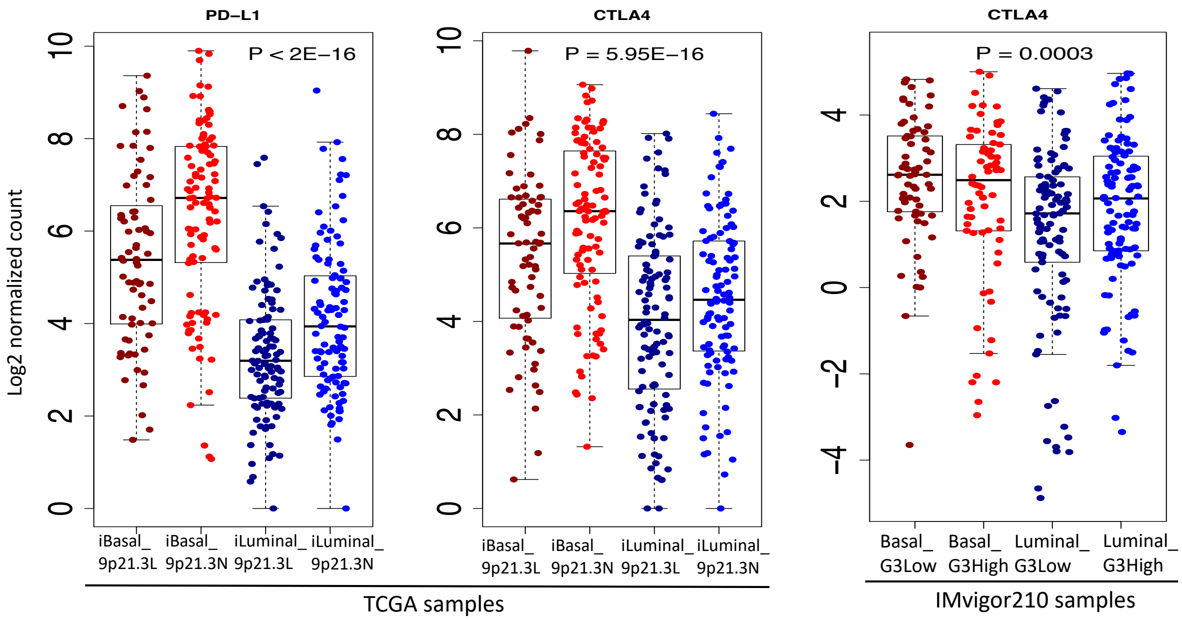

Supplementary Figure 7. *CTLA4* gene expression in the 4 subtypes of the TCGA and IMvigor 210 samples. G3Low/High: *MTAP/CDKN2A/2B* low/high expression. G3High/Low correspo nds to chr9 p21.3N/L, respectively. ANOVA was used for comparison among the subtypes

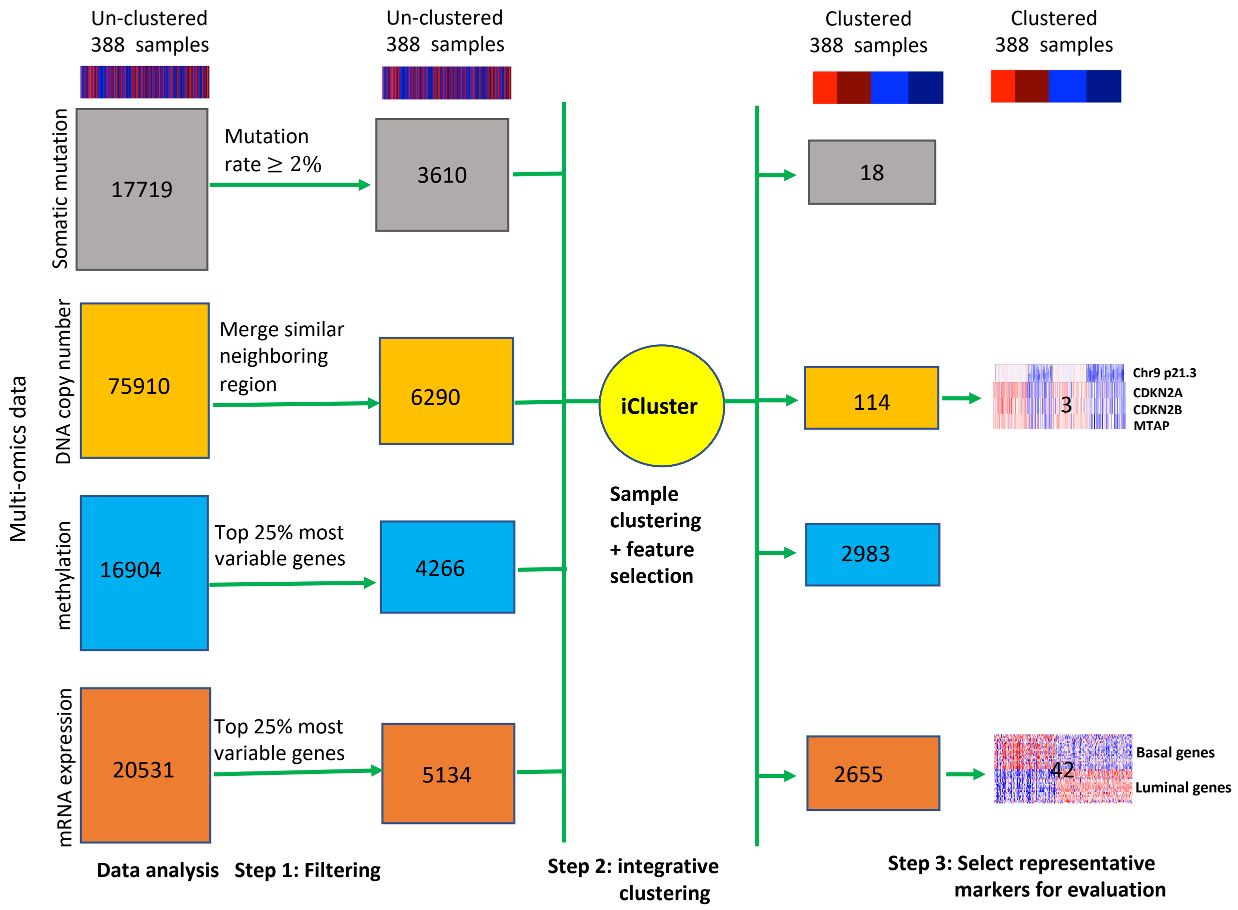

Supplementary Figure 8. Flowchart of the integrative clustering analysis of TCGA MIBC multi-omics data. The numbers of genomic features are shown in the rectangles.

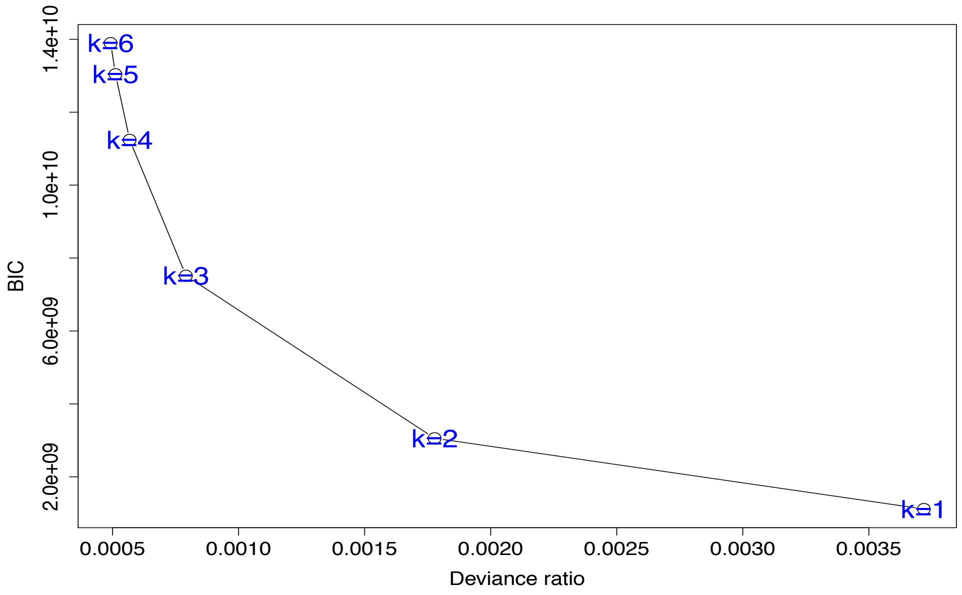

Supplementary Figure 9. Model fitting parameters of iClusterBayes. Bayesian information criterion (BIC) and deviance ratio for the cluster parameter k=1,2,3,4,5,6 are shown. For a given k, the samples can be divided into k+1 clusters. The BIC is minimum and deviance ratio is maximum when k = 1, suggesting that a 2-cluster solution is optimal.

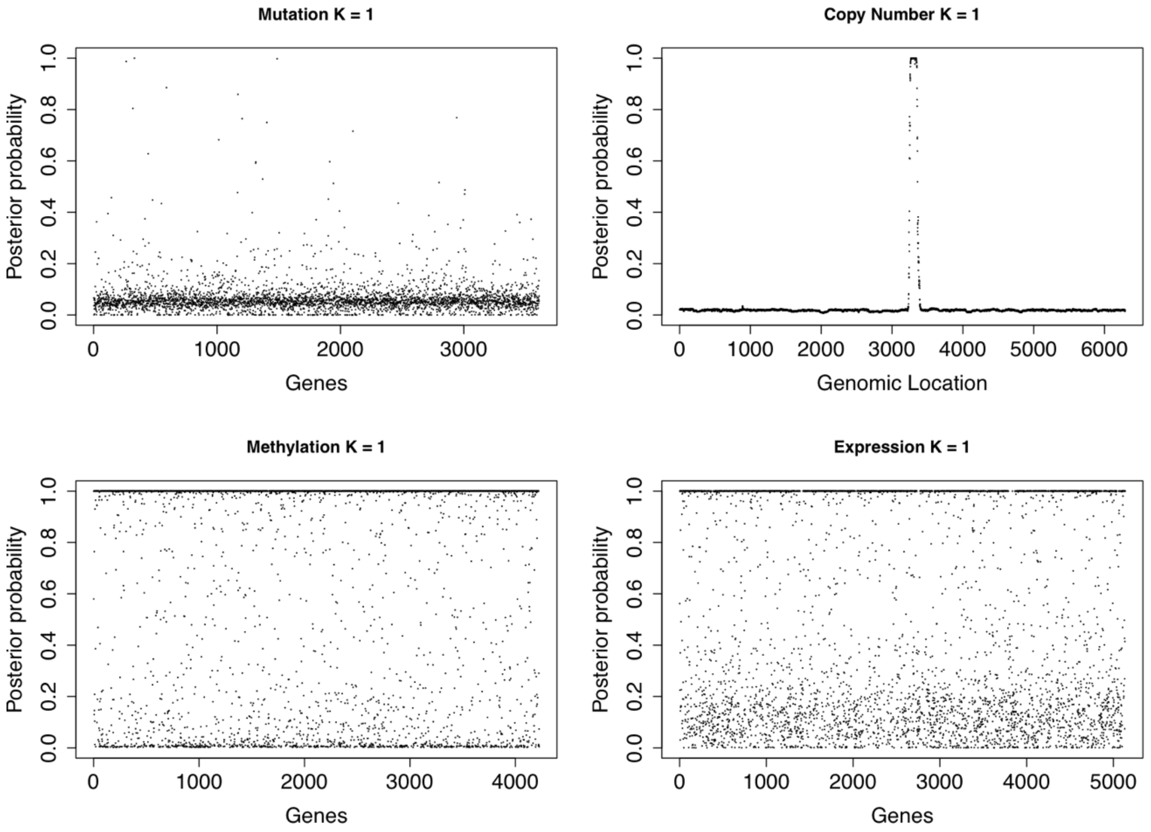

Supplementary Figure 10. Posterior probability of genomic features in the TCGA MIBC somatic mutation, DNA copy number, methylation and mRNA expression data sets for the 2-cluster solution (k=1).

Supplementary Table 1-2.

Supplementary Table 1. Patient characteristics in iBasal and iLuminal subtypes of TCGA MIBC data

Clinical variables	Level	iBasal	iLuminal	p-value
Pathologic stage	stage II*	41(33.61%)	81(66.39%)	0.011
	stage III	68(51.13%)	65(48.87%)	
	stage IV	63(48.09%)	68(51.91%)	
T stage	T2*	39(33.62%)	77(66.38%)	0.0017
	T3	101(54.01%)	86(45.99%)	
	T4	22(40%)	33(60%)	
M stage	m0	67(36.02%)	119(63.98%)	0.0078
	m1	6(54.55%)	5(45.45%)	
	mx	97(51.6%)	91(48.4%)	
N stage	n0	95(42.99%)	126(57.01%)	0.37
	n1	26(57.78%)	19(42.22%)	
	n2	30(41.1%)	43(58.9%)	
	n3	4(50%)	4(50%)	
	nx	14(38.89%)	22(61.11%)	
Gender	female	54(54%)	46(46%)	0.027
	male	118(40.97%)	170(59.03%)	
Radiation therapy	no	157(45.51%)	188(54.49%)	0.1
	yes	5(25%)	15(75%)	
History of neoadjuvant treatment	no	166(43.92%)	212(56.08%)	0.35
	yes	6(60%)	4(40%)	
Tobacco smoking history	no smoker	42(41.18%)	60(58.82%)	0.49
	smoker	124(45.26%)	150(54.74%)	
Years to birth	Median (Minimum - Maximum)	69 (43 – 90)	68(34 – 90)	0.4

Stage II: one patient in stage I was included into this category.
T2: one patient in TX and one patient in T1 was included into this category.

Supplementary Table 2. Multivariate cox regression analyses of iSubtypes and baseline clinical variables of TCGA MIBC data

Model 1

Variable	HR	95% CI-Low	95% CI High	p-value
iLuminal vs. iBasal	0.64	0.46	0.89	0.0086
Age > 70 vs. Age ≤ 70	1.44	1.05	2	0.026
Male vs. Female	0.75	0.53	1.06	0.11
Smoker vs. non-smoker	1.37	0.94	2	0.10
T3 vs. T2	1.73	1.15	2.58	0.0080
T4 vs. T2	2.53	1.51	4.23	0.00041

Model 2

Variable	HR	95% CI-Low	95% CI High	p-value
iLuminal vs. iBasal	0.62	0.45	0.85	0.003
Age > 70 vs. Age ≤ 70	1.5	1.1	2.05	0.011
Male vs. Female	0.84	0.6	1.19	0.33
Smoker vs. non-smoker	1.28	0.89	1.85	0.19
Stage III vs. II	1.52	0.97	2.37	0.067
Stage IV vs. II	2.67	1.75	4.05	4.41E-06
