# Supplementary material for: Integrative multi-omics analysis of muscle-invasive bladder cancer identifies prognostic biomarkers for frontline chemotherapy and immunotherapy
Source: Commun Biol. 2020 Dec 17;3:784. doi: 10.1038/s42003-020-01491-2 (PMC7746703; doi:10.1038/s42003-020-01491-2)
Supplement: Supplementary file 3 — Description of Supplementary Files [file 42003_2020_1491_MOESM3_ESM.pdf]

Description of Supplementary Data

File	Description
Supplementary_Information.pdf	Supplementary Figure S1-S10<br>Supplementary Table S1-S2
Supplementary_Data_1.xlsx	Data for Figure 1
Supplementary_Data_2.xlsx	TCGA MIBC clinical data for Figures 2 & 3E,3F
Supplementary_Data_3.xlsx	Data for Figures 3A, 3D
Supplementary_Data_4.xlsx	IMvigor210 clinical data for Figures 3B, 3C & Fig. 4
Supplementary_Data_5.xlsx	Data for Figure 5
Supplementary_Data_6.xlsx	Data for Figure 6
Supplementary_Data_7.xlsx	Data for Figure 7
